# Associations between morphokinetic parameters of temporary-arrest embryos and the clinical prognosis in FET cycles

**DOI:** 10.1515/med-2022-0592

**Published:** 2022-11-30

**Authors:** Hong-Xing Li, Yan Pang, Xiao-Ling Ma, Xue-Hong Zhang, Wen-qiang Li, Ya-Ming Xi

**Affiliations:** The First Clinical Medical College, Lanzhou University, Lanzhou 730000, China; Reproductive Medical Center, The First Hospital of Lanzhou University, Lanzhou 730000, China; Key Laboratory for Reproductive Medicine and Embryo of Gansu Province, Lanzhou 730000, China; PET-CT Center of Gansu Provincial Hospital, Lanzhou 730000, China

**Keywords:** embryo, pregnancy, morphokinetic parameter, frozen embryo transfer

## Abstract

Infertility is a major health concern worldwide. This retrospective study aimed to assess the predictive value of the morphokinetic parameters of temporary-arrest embryos for the pregnancy outcomes of women undergoing frozen embryo transfer (FET) cycles. In this study, we evaluated 244 FET cycles with 431 day-4 temporary-arrest embryos. They were categorized into two groups (pregnancy and non-pregnancy) according to the pregnancy outcomes of the women after embryo transfer on day 5, and their fundamental characteristics were compared. The morphokinetic parameters from the time-lapse monitoring system were assessed according to different pregnancy outcomes. The mean number of embryo blastomeres thawed on day 3 in the pregnancy group was 7.47, which was significantly higher than the number in the non-pregnancy group (*p* < 0.01). Besides, embryos in the non-pregnancy group contained more embryo fragments and lower grades than those in the pregnancy group (*p* < 0.001). Furthermore, morphokinetic parameters: tPNa, t2, t5, and t5_tPNf showed a statistical difference between the pregnancy and non-pregnancy groups (*p* < 0.05). Receiver-operating characteristic analysis revealed that the time from pronuclear fading to the 5-cell stage (t5_PNF) predicted the clinical prognosis outcomes (area under the curve = 0.64; 95% confidence interval [CI], 0.58–0.70; *p* < 0.001). The morphokinetic parameter t5_PNF could be regarded as a potential implantation predictor in our study.

## 1 Introduction

A major goal of *in vitro* fertilization (IVF)–embryo transfer (ET), an assisted reproductive technology, is to select embryos with the best potential for transplantation and to maximize the clinical pregnancy rate of patients. Frozen ET (FET) has been widely adopted as a conventional assisted reproductive technology. The success of FET depends on a variety of factors such as embryo developmental arrest (EDA). EDA is considered to result in IVF failure and is caused by abnormal preimplantation development, chromosomal abnormalities, and single-gene disorders [1,2]. However, our daily clinical and embryonic work found that some embryos exhibit a temporary developmental arrest in FET cycles. No embryo cleavage occurred within 24 h after day 3 (D3) thawing; meanwhile, the embryos had not developed to the compaction stage or blastocyst stage on day 5 (D5, 48 h after thawing). In this case, we define it as the D4 temporary arrest. The mechanism underlying temporary embryo arrest is unclear. Some FET cycles with temporary embryonic development arrest may lead to the cancellation of ET and others to lower pregnancy rates. The D4 temporary-arrest condition is causing confusion among clinicians and patients with low ovarian response, for they do not know whether those embryos can be transplanted or not.

Preimplantation genetic testing (PGT) and embryo biopsies provide a more direct evaluation of chromosome status and lead to increased embryo implantation and clinical pregnancy rates [3,4]. Nowadays embryo biopsy involves the removal of multiple trophectoderm cells at the blastocyst stage [5]. In practice, it has been found that some of the temporary-arrest embryos fail to develop to the blastocyst stage *in vitro*. Due to technical roadblocks and concerns regarding the long-term health of subsequent generations, the clinical application of PGT is limited. As shown in animal studies, embryo biopsy results in delayed formation of the blastocyst cavity and leads to an increased risk of neurodegeneration and dysfunction in subsequent generations [6,7,8]. Time-lapse morphokinetics (TLM) analyses have been increasingly used to predict developmental potential [9,10].

For decades, a morphokinetic grading system has been published by Meseguer et al. [11], which is considered one of the foundational studies of human embryo morphokinetics. So far, most of the published time-lapse studies are based on intracytoplasmic sperm injection (ICSI) fertilization rather than traditional IVF, mainly because it is impossible to know the exact time of sperm entry in the latter [12]. TLM is a tool for continuously observing embryonic development to obtain accurate embryo division time and cleavage pattern [13,14]. In this study, we retrospectively analyzed the value of the morphokinetic parameters of temporary-arrest embryos in predicting the pregnancy outcomes of women with FET who underwent ICSI.

## 2 Materials and methods

This retrospective, single-center cohort study used the medical records of infertile patients who underwent ICSI cycles followed by FET between September 2015 and January 2020.

### 2.1 Study group

Patients who underwent ET on D5 of the FET cycle were included in this study. On D3, the embryos were thawed and then cultured overnight. If the number of blastomeres did not increase on day 4 (D4), the embryo stage was defined as D4 temporary arrest. Then, the embryos were cultured for another day, and one to two embryos were transferred.

**Figure 1:**
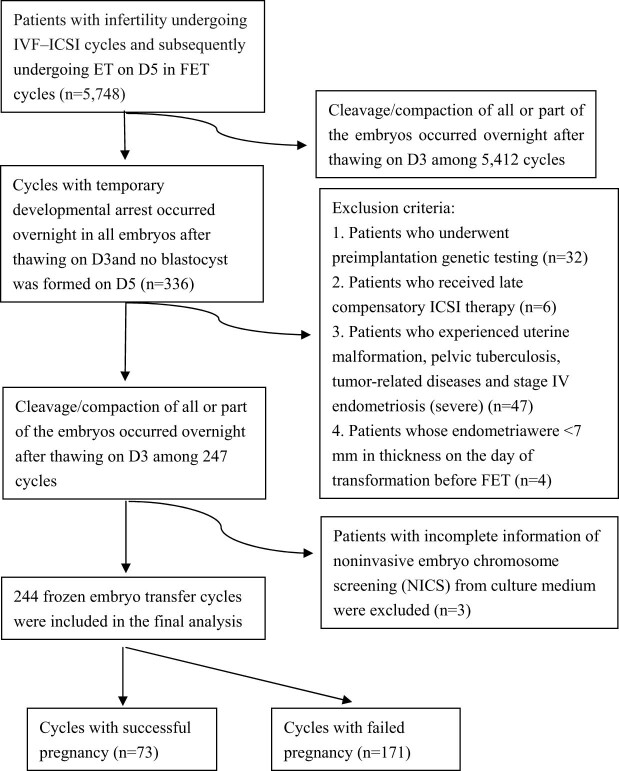
Flowchart showing the procedures used in selecting the study participants.

The exclusion criteria were as follows: (1) patients who underwent PGT; (2) received late compensatory ICSI therapy; (3) experienced uterine malformation, pelvic tuberculosis, tumor-related diseases, or stage IV endometriosis (severe); and (4) had an endometrial thickness of <7 mm on the day of transformation prior to FET. Finally, 244 FET cycles were selected for the final analysis from a total of 5,748 cycles. These 244 cycles included 73 cycles of successful pregnancy and 171 failed cycles, as shown in Figure 1. In this study, we evaluated 244 FET cycles with 431 D4 temporary-arrest embryos. They were categorized into two groups (pregnancy and non-pregnancy) according to the pregnancy outcomes of the women after ET on day 5.

### 2.2 Ovarian stimulation

In long or short protocols, the patients received controlled ovarian stimulation with recombinant follicle-stimulating hormone (FSH) (Puregon®; Organon, the Netherlands) and gonadotropin-releasing hormone agonists (Decapeptyl®; Ferring, Germany). FSH doses were changed according to the ovarian response, which was assessed using ultrasound and by evaluating estrogen and progesterone levels. Then, 5,000–10,000 units of human chorionic gonadotropin (Pregnyl®; Saint-Prex, Switzerland) were administered to induce oocyte maturation. At 36 h after induction, follicular aspiration was performed under ultrasound guidance.

### 2.3 Embryo culture

ICSI was performed to fertilize all embryos in accordance with the manufacturer’s instructions. On the first day (16–18 h after insemination), the fertilization status was measured and the appearance of two polar bodies and two prokaryotic cells was observed. The embryos were cultured in time-lapse incubators (Embryoscope; Unisense‑FertiliTech, Denmark) at 37°C, 6% CO_2_, and 5% O_2_ with Vitrolife G-I plus cleavage medium (Vitrolife, Goteborg, Sweden). The Gardner grading method was used to evaluate embryo development on the third day after insemination [3]. All embryos were cultured for 3 days before vitrification using the VitriFreeze™ Media Kit (Kitazato, Japan). All samples were stored in liquid nitrogen. In the FET cycles, D3 cleavage embryos were thawed using the VitriThaw™ Media Kit (Kitazato) and cultured overnight in Vitrolife G-II plus.

### 2.4 Morphokinetic assessment of embryo early development

All the embryos cultured in the time-lapse were observed and annotated on day 3 by one embryologist (L.H.) using the Vitrolife Embryoviewer software. In this study, the morphokinetic parameters include the following: tPNa (appearance of two pronuclei); tPNf (both pronuclei faded); t2, t3, t4, and t5 (time between intracytoplasmic injection and 2-, 3-, 4-, and 5-cell stages, respectively); CC2 (length of the second cell cycle [t3–t2]); CC3 (length of the third cell cycle [t5–t3]); and t5_tPNf (time from pronuclear fading to 5-cell stage). Traditional morphological assessment has also been carried out based on embryo images at 68 h post-fertilization according to previously published criteria [15].

### 2.5 Endometrial preparation in frozen cycles

#### 2.5.1 Replacement with artificial hormones

The cycle began on day 3, with patients taking orally 2–6 mg of Estrace, Allergan’s estradiol, for endometrial preparation. Whether the endometrium was ready for the ET procedure was assessed under endometrial ultrasound. Weekly measurements of endometrial thickness were performed until it was >7 mm. According to the recommended defrost and ET days, the women were injected vaginally with 200 mg of progesterone suppository three times a day to initiate luteal support. On the third day of progesterone administration, the embryos were defrosted and incubated overnight.

#### 2.5.2 Natural cycles

Spontaneous menstruation was followed by regular monitoring of endometrial thickness and follicular development under a series of ultrasound steps. Progesterone and luteinizing hormone (LH) levels were measured until the LH level reached its peak value on the day prior to oocyte ovulation (at which the LH level was >180% of the baseline level). Three daily injections of 200 mg of progesterone suppositories began to show an effect on the second day after progesterone administration. On the third day after progesterone administration, the embryos were defrosted.

### 2.6 ET and confirmation of implantation

During the natural cycle or hormone replacement FET cycle, one or two embryos were thawed and incubated overnight for subsequent transplantation, depending on the age of the patient. All ET procedures were performed using an ultrasound-guided standardized technique. Clinical pregnancy was confirmed using fetal heartbeat ultrasound at 7 weeks of gestation.

Pregnancy rate = number of clinical pregnancies/number of transplantation cycles.

Implantation rate = total number of implanted embryos/total number of transferred embryos.

### 2.7 Statistical analysis

IBM SPSS v.22 was employed to analyze the data. The Student’s *t-*test was adopted to compare the continuous variables that were represented by mean values ± SD, and the chi-square test was adopted to compare the categorical variables. When a *p*-value was <0.05, the comparative result was considered to indicate a statistically significant difference.


**Ethics approval:** This study was approved by the Ethics Committee of the First Hospital of Lanzhou University (LDYYLL2019-42).
**Consent to participate:** The authors certify that they have obtained all appropriate patient consent forms. The patients have given consent for their clinical information to be reported in the journal. The patients understand that their names and initials will not be published and due efforts will be made to conceal their identity, but anonymity cannot be guaranteed.

## 3 Results

### 3.1 Demographic data and cycle features

A total of 235 controlled ovarian hyperstimulation cycles that were subjected to ICSI fertilization were included in the study. The clinical pregnancy rate and implantation rate for FET cycles with temporary developmental arrest were 29.92% (73/244) and 21.58% (93/431), respectively. No significant differences were found between the pregnancy and non-pregnancy groups in baseline characteristics and demographic data, including age, body mass index, anti-Müllerian hormone level, serum estradiol and progesterone levels on trigger day, number of metaphase II oocytes retrieved, and endometrial thickness on transplantation day (Table 1).

**Table 1:** Demographic characteristics

	Pregnancy group	Non-pregnancy group	*p*-value
Total number of controlled ovarian hyperstimulation cycles	71	164	NS
Total number of FET cycles	73	171	NS
Age	32.79 ± 4.82	33.38 ± 6.39	NS
Body mass index (kg/m^2^)	21.64 ± 3.12	22.10 ± 3.52	NS
AMH level (ng/mL)	3.78 ± 2.61	3.49 ± 2.92	NS
E2 level on trigger day (pg/mL)	2869.95 ± 1072.21	2934.50 ± 1191.68	NS
Stimulation duration (days)	11.33 ± 1.62	11.56 ± 1.84	NS
P4 level on trigger day (ng/mL)	1.03 ± 0.43	0.98 ± 0.42	NS
Number of oocytes retrieved	14.25 ± 6.83	13.67 ± 5.29	NS
Number of MII oocytes retrieved	12.11 ± 5.38	11.61 ± 5.47	NS
Endometrial thickness (mm)	0.98 ± 0.21	1.00 ± 0.19	NS

Note: Values are presented as numbers, mean ± SD, or %. AMH: anti-Müllerian hormone, E2: serum estradiol, P4: serum progesterone, MII: metaphase II.

### 3.2 Comparison of embryo morphology

A total of 431 transplanted embryos were annotated retrospectively into two groups according to whether pregnancy occurred from the FET cycles. Table 2 shows a comparison of embryo morphology between the two groups, including the mean number of embryo blastomeres on D3 and embryo ratings and fragments after D3 thawing. The mean number of embryo blastomeres thawed on D3 in the pregnancy group was 7.47, which was remarkably more than the number in the non-pregnancy group (*p* < 0.01). In addition, embryos in the non-pregnancy group had more embryo fragments and lower grades than those in the pregnancy group (Table 2), which may indicate lower developmental potential.

**Table 2:** Comparison of embryo morphology

	Pregnancy group	Non-pregnancy group	*p*-value
Total number of FET cycles	73	171	NS
Total number of embryos transferred	93	338	NS
Number of embryos transferred in FET cycles	1.80 ± 0.30	1.71 ± 0.46	NS
Mean number of embryo blastomeres on D3	7.47 ± 2.32	5.64 ± 2.23	<0.01
Embryo ratings after D3 thawing^a^			
Grade I	7	0	
Grade II	68	135	
Grade III	18	203	
Embryo fragments after D3 thawing (%)^b^			
<10	30	36	
10–20	51	114	
20–30	12	188	

Note: Values are represented by numbers, mean ± SD, or %. ^a^ *p* < 0.001, *χ* ^2^ = 66.05, ^b^ *p* < 0.001, *χ* ^2^ = 59.41.

### 3.3 Comparison of the TLM parameter between pregnancy and non-pregnancy groups

A total of 431 embryos were annotated on day 3 using the Embryoviewer software (Table 3). Among those TLM parameters, tPNa, t2, t5, and t5_tPNf showed statistical differences between the pregnancy and non-pregnancy groups (*p* < 0.05). Receiver-operating characteristic analysis revealed that the time from pronuclear fading to the 5-cell stage (t5_PNF) predicted the clinical prognosis outcomes of all embryos (area under the curve = 0.64; 95% CI, 0.58–0.70; *p* < 0.001). The morphokinetic parameter t5_PNF could be regarded as a potential implantation predictor in our study.

**Table 3:** Comparison of time-lapse parameter

TLM parameter (*n*)	Pregnancy group (*n* = 93)	Non-pregnancy group (*n* = 338)	*p*-value
tPNa	8.7 ± 2.3	9.43 ± 3.0	0.018
tPNf	24.5 ± 2.9	24.7 ± 3.3	NS
t2	26.8 ± 2.9	27.4 ± 3.1	0.033
t3	36.9 ± 4.5	37.6 ± 4.7	NS
t4	38.9 ± 4.7	39.4 ± 4.4	NS
t5	48.8 ± 7.2	50.1 ± 7.3	0.006
CC2	10.4 ± 3.2	10.5 ± 3.0	NS
CC3	12.5 ± 4.7	12.6 ± 4.1	NS
t5_tPNf	24.2 ± 6.3	25.6 ± 8.6	<0.001
t5_t2	22.3 ± 6.3	22.5 ± 6.2	NS

Note: Values are represented by mean ± SD.

## 4 Discussion

In daily clinical and laboratory work, cleavage-stage embryos may undergo temporary developmental arrest on D4 and cannot develop into blastocysts on D5/6 in FET cycles, especially for patients with low ovarian response. In such instances, patients are not sure whether they should undergo ET.

Patients who underwent ET on D5 in FET cycles were included in the present study. On D3 after treatment, the embryos were thawed and then cultured overnight. If the number of blastomeres did not increase on D4, the embryo stage was defined as D4 temporary arrest. Then, the embryos were cultured for another day, and one to two embryos were selectively transferred. Abnormal preimplantation development, chromosomal abnormalities, and single-gene disorders are thought to be factors resulting in EDA [1,2]. Embryo freezing and thawing operations during the IVF/FET cycle may affect the rate of embryo development, resulting in D4 temporary arrest [16].

In this study, 244 FET cycles were selected for final analysis. Of these, 73 cycles resulted in successful pregnancies, and there were 171 failed cycles, suggesting that temporary-arrest embryos had certain developmental potential. To the best of our knowledge, this is the first report on the outcome of the transfer of temporary development-arrested embryos on D4 in FET cycles. The clinical pregnancy rate and implantation rate for thawed embryos with temporary developmental arrest were 29.92% (73/244) and 21.58% (93/431), respectively, which were lower than the published data of our center and other centers [17]. Based on the data analysis, ET should not be abandoned for those D4 temporary-arrest embryos, and there was still the possibility of successful implantation.

It is internationally recognized that TLM can be used to screen embryos by annotating the timing parameters, which could improve the clinical pregnancy rate of ET. In our study, four morphokinetic parameters: tPNa, t2, t5, and t5_tPNf showed a statistical difference between the pregnancy and non-pregnancy groups (*p* < 0.05). The timing parameter t5_tPNf had been regarded as a major implantation predictor in previous publications [11,13]. In the time-lapse selection model described by Liu et al. [13], if an embryo had t5_tPNf between 24.67 and 28.01 h, it could be categorized as grade A; otherwise, if t5_tPNf <24.67 h, it could be categorized as grade A+. Implantation rates of grade A and A+ embryos were 52.9% and 36.1%, respectively. Our data showed that the parameter t5_tPNf was 24.2 ± 6.3 h for the pregnancy group and 25.6 ± 8.6 h for the non-pregnancy group, respectively (*p <* 0.001), suggesting that t5_tPNf may be a significant implantation predictor for D4 temporary-arrest embryos. In conclusion, embryos that grow slowly still have the potential to implant, even those with a temporary arrest after thawing.
